# Barriers and Facilitators to Early-life Wildfire Smoke Protection in a Rural Population: The Role of Community in Research and Public Health Practice

**DOI:** 10.21203/rs.3.rs-7359173/v1

**Published:** 2025-09-22

**Authors:** Javier Silva, Aileen Andrade-Torres, Corbin Schuster, Jessica Black, Jari Tavira, Virginia Yelechin, Aaliyah Villa, Karolynn Tom, Shelby Clark, Catherine J. Karr, Christine T. Loftus

**Affiliations:** University of Washington; University of Washington; Oregon State University; Heritage University; Heritage University; Heritage University; Heritage University; Yakima Valley College; Heritage University; University of Washington; University of Washington

## Abstract

**Background:**

Millions of U.S. families are exposed to unhealthy levels of wildfire smoke (WFS) annually. Interventions to reduce the impacts of WFS on child health are urgently needed, especially for families in vulnerable communities, where WFS exposure is high yet resources and opportunities for self-protection are limited. Little is known about parents’ current engagement in protective behaviors, a knowledge gap that challenges development of feasible and acceptable interventions. We aimed to address this gap in a rural community in Washington State with high concerns about WFS and child health.

**Methods:**

We conducted community meetings and utilized an existing framework of behavioral change to draft a conceptual model of barriers and facilitators of WFS protective actions. Using this model, we identified hypothesized determinants of protective behaviors (N = 11) that could be characterized by surveying parents about perceptions of WFS and experiences in past fires. Surveys were administered by local college students at community events and online, in English and Spanish. Linear regression with robust standard errors estimated associations between z-scores of determinants of action and total action score, a sum of frequencies of protective actions (N = 6) taken in past smoke events. Predictors of frequencies of each specific action were also characterized in exploratory analyses.

**Results:**

Of N = 199 participating parents, 84.8% were Hispanic, 55.3% were born in Mexico, and 58.8% had an annual income < 40K. The most frequent protective action was staying indoors, and the least was leaving the region, with 54.8% and 1.5% reporting doing so “often”, respectively. Impacts on child health in past WFS events and observations of other community members taking action were most strongly associated with action scores (ß=0.91 [95% CI: 0.50, 1.31] and 0.67 [95% CI: 0.16, 1.18] per standard deviation, respectively). Chronic health conditions, general WFS knowledge, and practical knowledge about WFS mitigation also predicted more actions.

**Conclusions:**

These novel data identified facilitators of protective behaviors that that can be targeted in future interventions. The observed importance of social norms is consistent with community members’ descriptions of a local culture of *familismo*, prevalent in Hispanic and immigrant populations, and a strength that WFS interventions should leverage.

## 1. INTRODUCTION

Fueled by a warming climate, the intensity of wildfires in the Western US. is increasing, resulting in a substantial rise in community exposures to wildfire smoke (WFS).^1^ Prenatal and early-life WFS exposure can have significant and lasting health impacts on child health and well-being.^2–5^ Particulate matter of 2.5 microns or less in aerodynamic diameter (PM_2.5_) is the primary toxic component of WFS and has been linked to numerous adverse maternal and child health outcomes.^1,6^ Upon inhalation, PM_2.5_ penetrates the lower respiratory tract and increases local airway inflammation. Inhaled particles can also translocate into the bloodstream and cross the blood-brain barrier, causing systemic health effects across multiple organ systems.^7^ When pregnant women are exposed, PM_2.5_ may pass the placental barrier and directly interfere with healthy fetal development through several mechanisms, such as increased production of reactive oxidative species, immune system disruption, and/or epigenetic modifications.^8^ Notably, studies in animal models and human populations suggest that WFS-specific PM_2.5_ may be even more harmful than PM_2.5_ from other sources, such as traffic.^9^ Epidemiological studies in a variety of settings report associations between prenatal and early-life wildfire PM_2.5_ exposure and adverse birth outcomes, childhood respiratory infections, and respiratory symptoms and hospitalization for children with asthma.^5, 10–11^

These increases in WFS could widen existing child health disparities in the US. In many parts of the US, including Washington State, the communities facing the highest WFS exposures have a relatively high proportion of low income and minoritized individuals.^12–14^ Recent studies also suggest that people of vulnerable sociodemographic groups may be more susceptible to adverse effects of WFS than others, even at the same level of exposure.^14–15^ Furthermore, families living in remote, low-resourced regions and those with socioeconomic adversity face additional barriers to protecting themselves and their children from WFS, including limited access to materials, information, and programs supporting protective behaviors.^12, 16–17^

Interventions to reduce WFS exposure are urgently needed, especially those aimed at protecting children in the most vulnerable communities. Common public health recommendations for reducing WFS health risks includes using indoor air cleaners, staying inside, wearing N95 masks, consulting outdoor air quality data, talking with health care providers, and others.^4,18^ However, little is known about the current practice of these protective behaviors or the barriers to taking actions.^19^ A better understanding of these factors is essential to develop effective, community-specific intervention protocols and resources about the impacts of WFS on child health. Our group and others have conducted successful intervention research on childhood air pollution exposure in remote, low-resource settings, but studies of WFS-specific interventions are lacking.^20–22^

We therefore aimed to address these knowledge gaps in a rural, low-resource community where children are at high risk of WFS-related health effects. Our objectives were to characterize parents’ attitudes, perceptions and knowledge pertaining to WFS and describe engagement in protective actions. We further aimed to analyze the facilitators of and barriers to protective behaviors by applying the Risks, Attitudes, Norms, Abilities and Self-regulation (RANAS) model of behavior change.^23^ Ultimately, the results of this study will be informative for development of future interventions for our study population and similar regions across the Western US.

## 2. METHODS

### 2.1 Study design and setting

**Figure 1:**
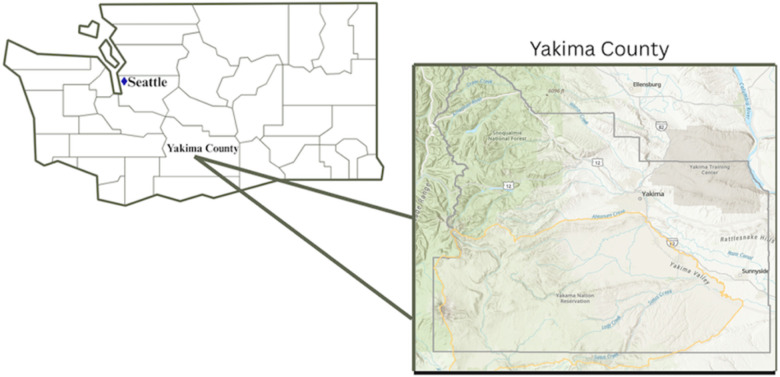
Map of the Yakima Valley. All study activities, including enrollment of participants and data collection, took place in the Yakima Valley, a rural and agricultural region of Yakima County spanning from the city of Tieton to the town of Sunnyside. The primary academic partner was the University of Washington, in Seattle, about 150 miles away. Surveys were collected at in-person events in the four cities labeled on the map.

This cross-sectional study was set in the Yakima Valley, a region of central Washington State known for its robust agricultural economy and rural landscape (Fig. 1).^24^ The surrounding Yakima County experiences frequent WFS events – particularly in the summer and autumn – which significantly degrade air quality and pose health risks to residents.^25^ The growing frequency of WFS episodes is worsening the longstanding air quality issues in this region, caused in part by prevalent agricultural burning practices and aggravated by winter inversion conditions, in which cool air in the upper atmosphere trap a blanket of air below, effectively concentrating air pollution near ground level.^26,27^ For these reasons, levels of outdoor PM_2.5_ in the county are amongst the highest in the U.S. Recent estimates indicate that Yakima County PM_2.5_ is about 50% higher than the nationwide county average.^28^

The region is home to a diverse population, including a high proportion of Spanish speakers. More than 40% of Yakima County residents identify as Hispanic or Latino, and this proportion is higher in the Yakima Valley.^29^ Some Spanish-speaking residents experience linguistic isolation, which can impact a family’s ability to access and comprehend health-related information, including public communications related to wildfire smoke exposure. The Yakima Valley has a significant number of first-generation immigrants, many of whom originate from Mexico and are employed in the agricultural industry. These individuals often face unique challenges related to health literacy as well as ability to take preventive measures due to language barriers, limited access to healthcare, and financial constraints.^30^ There is also a high burden of chronic diseases in the region, contributing to increased vulnerability to WFS exposure. For example, adults in the lower Yakima Valley have approximately 50% higher rates of obesity, diabetes, and high blood pressure compared to Washington State.^28^

### 2.2 Community-engaged approach

#### 2.2.1 Overview and rationale.

The application of community-engaged research (CEnR) methods can improve adoption of health behaviors and address community concerns around health literacy, particularly among underserved and vulnerable communities.^31–32^ Many approaches to CEnR have been successfully applied in environmental health research, as summarized in recent reviews.^32–33^ We designed a CEnR approach tailored to this study’s research objectives that leveraged decades of research collaborations between UW, Heritage University and community partners,^21, 34–37^ and responded directly to community requests for more training and research experiences for local students. Specifically, we: 1) engaged a community advisory board (CAB), because a CAB can help understand the barriers and facilitators of health behaviors, support academia-community collaborations, and encourage bidirectional learning; and 2) directly involved local college students in data collection, interpretation of findings, and dissemination of results, to provide valuable career-building research opportunities and to increase the community’s trust of the research team.^38^

#### 2.2.2 Study Community Advisory Board.

**Figure 2:**
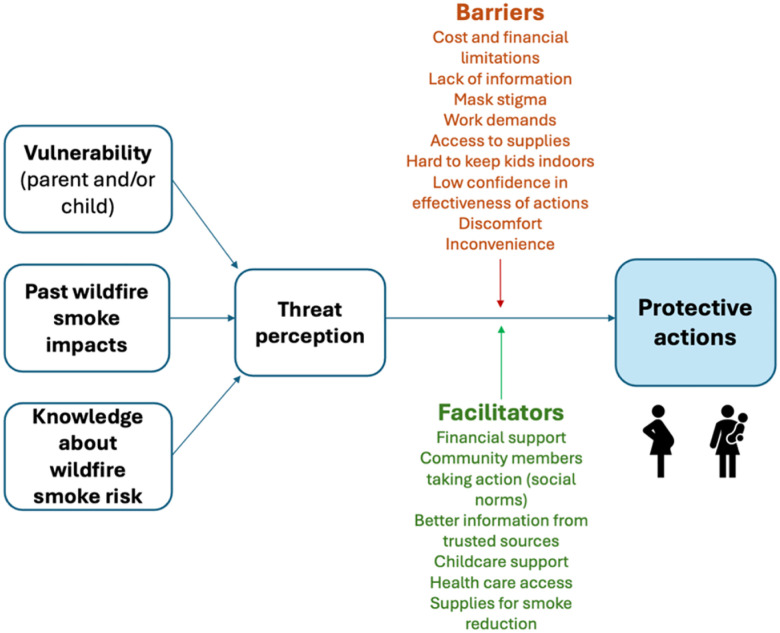
Conceptual model of barriers and facilitators to protective actions. Based on feedback from a community advisory board, we drafted a conceptual model for the factors that lead to the decision of a parent to take action to reduce wildfire smoke exposure, as well as the barriers and facilitators to action for individuals who perceive threat.

We assembled a CAB comprised of five long-term Yakima Valley residents employed in health care, higher education, childcare, public health and community programs. All were parents, worked with children (e.g., in an educational context), and/or coordinated community programs supporting local families. We convened two CAB meetings at the start of the study to inform a conceptual model, advise on research protocols, and review surveys and other data collection materials. At the first meeting, the CAB was asked about their cumulative experiences with wildfire smoke in the Yakima Valley, including their perceptions of health risks, observations about protective actions taken by parents and other community members in the region, specific challenges people face in protecting themselves and children, and ideas for future resources related to wildfires smoke that would be acceptable and effective in the Yakima Valley community. These insights were used to draft a preliminary model of barriers and facilitators of WFS-protective actions in this community (Fig. 2), reviewed and revised at a second CAB meeting. CAB members also piloted and gave feedback on the first survey draft, including English and Spanish versions.

#### 2.2.3 Local community members for data collection.

Interns were recruited from Heritage University, a Hispanic-Serving Institution (HSI) and Native American Serving Non-Tribal Institution (NASNTI) in Toppenish, Washington. Selected interns were local to the Yakima Valley and represented diverse academic majors including biology, environmental science, and nursing. The interns were mentored by two public health graduate students at UW, one of whom also originated from the Yakima Valley, providing additional guidance grounded in both academic expertise and local knowledge.

Interns assisted with identifying appropriate sample locations based on local knowledge of community gathering points, especially those attended by parents and young families, such as summer school lunch distribution programs. This approach facilitated meeting potential participants at locations they already frequented. Prior to fieldwork, interns completed training in human subjects’ research, health information privacy and security for clinical investigators, social and behavioral best practices for clinical research, community engaged and community-based participatory research, and outdoor heat safety. In addition, interns participated in a structured seminar series on environmental health research in agricultural communities led by study investigators.

For data management, interns were trained in the Research Electronic Data Capture (REDCap) platform and handled data entry, identified missing data points, and conducted quality assurance checks. All interns were bilingual, facilitating efficient processing of data collected in both English and Spanish.

### 2.3 Survey Development

#### 2.3.1 Overview.

We aimed to develop a survey in English and Spanish to be self-administered online or in-person, with an average completion time of 15 minutes. Survey topics include participant characteristics (e.g., sociodemographics and health behaviors), protective behaviors taken in past smoke events, opinions about the need for specific future community resources, and potential determinants of protective behavior as guided by conceptual models, described below. We conducted a review of the literature for existing instruments but identified none that could be directly utilized for our research objectives.

#### 2.3.2 Measurement of hypothesized predictors of protective behaviors.

Understanding motivators of health behaviors requires examining the nuanced psychological and social factors that influence an individual’s decision to take protective actions during wildfire smoke events. We adapted the Risks, Attitudes, Norms, Abilities, and Self-regulation (RANAS) model as a guiding model for these factors.^23^ This framework has been successfully applied in prior environmental health research to demonstrate how cognitive, emotional, contextual determinants shape health-related decision-making and behavioral adoptions. Studies by Flanagan et al.^39^ in Central Maine and VanDerGeest et al.^40^ in Yakima Valley both applied the RANAS model to investigate drivers of testing well water for arsenic and nitrate contamination, respectively. The RANAS model was also applied to investigate lived experiences and personal intentions that motivate preparedness and protective actions following extreme weather events in the Republic of Ireland.^41^

To direct survey development, we selected N = 11 potential barriers and facilitators of WFS self-protection that were relevant to future WFS interventions and feasible to ascertain by survey. N = 9 predictors were chosen to represent one or more RANAS factors in each of four blocks of the framework: Risks, Attitudes, Norms, and Ability. (No RANAS Self-regulation factors were assessed due to lack of relevance to this context.) These predictors were: general WFS knowledge, estimated *via* responses to questions that assessed knowledge of health risks and mask efficacy; child health and parent health vulnerability, defined as existing child and parent chronic health conditions, respectively (two separate variables); perceived threat to child health, assessed as parent’s perceived threat of WFS to child health; perceived expense of keeping one’s home free of WFS; whether other community members took protective actions in smoke episodes; practical, “how to” knowledge about WFS exposure reduction; confidence in one’s self to effectively reduce WFS exposure; and financial resources, assessed as total household income. We ascertained two additional factors, to represent adverse prior experiences with wildfire smoke events because this was a predictor in the conceptual model developed with the CAB (Fig. 1): the severity of child health problems in past WFS events and the count of total symptoms experienced by child(ren) and/or the parent in past WFS events.

In addition to these N = 11 predictors, we estimated variables corresponding to several predictors evaluated in secondary analysis of specific protective actions (Sect. 2.5): mask-specific knowledge, feelings about masks, stigma about mask wearing, confidence to use an indoor air cleaner, confidence to look up local air quality, and confidence to help child(ren) use a mask.

See Supplemental Table 1 for a description of the specific RANAS factors addressed by each predictor, survey questions used to assess each predictor, and method for calculating the corresponding analytic variables.

#### 2.3.3 Pilot testing.

Both English and Spanish versions of the survey were tested extensively by individuals on the CAB and the research team (N = 13), for length, clarity, face validity, and content validity (e.g., whether response categories adequately covered the range of individual experiences during WFS events). Spanish translations were conducted by a research coordinator who is a native Spanish speaker and additionally reviewed by members of the CAB and research team who were current or past Yakima Valley residents (N = 12) for accuracy of local dialect and cultural relevance. Surveys were tested on paper and electronic versions programmed into REDCap and evaluated on a variety of devices, including smartphones and computers.

### 2.4 Participant enrollment and data collection protocols

#### 2.4.1 Human subjects

Research was conducted in compliance with relevant laws and institutional guidelines. The study was determined to be exempt by the University of Washington and Heritage University institutional review boards (IRBs). Privacy rights of human subjects were observed. All participants provided informed consent and were offered a financial incentive for participation.

#### 2.4.2 Eligibility criteria

Inclusion criteria were: 18 years of age or older, residence in the Yakima area for at least 2 years, proficiency in English and/or Spanish, and either currently pregnant and/or a parent of at least one child aged 12 years old or younger. Individuals were ineligible if another household member had already participated in the study.

#### 2.4.3 Recruitment and survey administration

All data collection was conducted between June and September of 2024. Participants were recruited at in-person data collection events and through flyers posted in community locations and shared *via* social media platforms. In-person data collection occurred at community events across the Yakima Valley likely to be attended by parents and pregnant women, including school events (e.g., distribution of meals for families receiving free and reduced priced lunch benefits), local health fairs, and open hours at food banks. Participants could also complete surveys online, and one participant completed the survey by phone. Participants elected to take the English or Spanish version based on preference.

#### 2.4.4 Data management

Data collected on paper were entered into a REDCap database by a study team member and reviewed by a second research assistant for accuracy; discrepancies were resolved through discussion, including adjudication by additional team members if necessary. Responses that were illegible or internally inconsistent were infrequent (< 1% of data elements collected, across all surveys) and were marked as missing.

### 2.5 Statistical analyses

All analyses were conducted using Stata version 13.1 (StataCorp, College Station, TX USA). We conducted descriptive analyses of all variables collected, summarizing distributions across the sample as mean and standard deviation (continuous variables) or as counts and percentages (categorical variables). Variables corresponding to each hypothesized predictor of protective behaviors were calculated using one or more survey items. Factors derived using multiple questions or endorsement of multiple response categories included parent existing health conditions, child existing health conditions, general WFS knowledge, “how to” WFS knowledge, WFS impact on general child health, and parent/child symptoms in past WFS events (Supplemental Table 1). No validated approaches to calculating these specific measures exist in the literature, to our knowledge; therefore, we determined the approach for each based *a priori* on expert judgment and examples from other studies. Predictor variables were each standardized to the analytic sample distribution through conversion to a z-score, to aid in interpretability and for between-predictor comparisons. Pairwise correlation between all predictors was calculated as Pearson correlation coefficients. We also calculated a total protective action score for each participant as the sum of responses (never = 0, sometimes = 1, often = 2) to questions about frequency of engagement in six protective behaviors in past WFS events: staying indoors, checking the air quality index (AQI), mask wearing, talking to a doctor or other health care provider, using an indoor air cleaner, and leaving the region for a place with cleaner air.

We estimated associations between predictors and total protective action score using linear regression with robust standard errors. For our primary analyses, we estimated associations between each standardized factor and total protective action score in bivariate analyses. Two sensitivity analyses were conducted: 1) we estimated associations adjusted for all other factors by including all in one regression model, as covariates, and 2) we used stepwise backwards selection for covariate selection in a regression model, with a threshold of 0.20 for variable selection. Finally, we analyzed the responses to each protective action (never = 0, sometimes = 1, often = 2) as secondary outcomes, estimating associations between each predictor and behavior-specific frequency separately. For example, the analysis of mask wearing frequency included additional factors specific to mask usage, including reported stigma about mask wearing, perceptions about expense specific to mask wearing, feeling about wearing masks, knowledge specific to mask usage, and confidence to help their child(ren) correctly wear masks.

## 3. RESULTS

### 3.1 Description of study population

A total of 199 participants participated in this study (Table 1). Of these, 22 were male (11.1%) and 176 were female (88.4%). The mean age of study participants was 36.2 years (SD = 8.7 years). About a third of participants (37.1%) reported having less than a high school education, 30.2% had a high school degree, and 30.7% had completed at least some college-level education. For reported race and ethnicity, 84.8% of participants identified as Hispanic or Latino, 9.5% as White, and 5.1% as American Indian or Alaskan Native. Smaller groups included Black or African American, Middle Eastern or North African, and Asian communities, collectively comprising 5.5% of the study sample. Most participants (71.4%) reported residing in the region for 10 or more years. The distribution of reported household incomes included 58.8% with an income under $40,000, 28.2% between $40,000-$75,000 and 6% above $75,000. Most families received public assistance: food stamps or supplemental nutritional assistance program (SNAP) benefits (41.4%), the women, infants and children (WIC) nutrition program (37.4%), and free or reduced lunch school program (60.6%).

Participants were asked about existing health, occupational exposures to WFS, and the home environment (Table 2). Parents’ most common health conditions included anxiety or depression (26.1%), allergies related to lungs, eyes, or nose (22.6%), and asthma (16.6%). The most common child health conditions included allergies (39.3%), asthma (29.6%), and eczema (21.4%). A small proportion of respondents reported being a smoker (4%), and 10.7% had a smoker in the household. Many participants (37.4%) had jobs requiring outdoor labor; of those, 44.4% and 36.1% work outdoors approximately 4–8 hours or over 8 hours a day, respectively, during wildfire seasons.

### 3.2 Perceptions of wildfire smoke, knowledge, and past experiences

Participants demonstrated knowledge about WFS health risks and methods for reducing exposure in a series of true/false questions, plus questions about relative mask effectiveness (Supplemental Figs. 1 and 2). The composite general knowledge score ranged from 0 to 10, with a mean (SD) of 6.2 (2.1); the “how to” knowledge score ranged from 0 to 4 and had a mean (SD) of 2.2 (1.3). Overall, this population perceived WFS to be a serious threat to child health, with 57.7% describing it to be an “extreme threat” and 21.9% “very much a threat” (Table 3). We observed a range in parents’ confidence to reduce household exposures to wildfire smoke, with only 26.6%, 29.4% and 41.9% of participants feeling “very confident” to effectively set up and use a portable air cleaner, to check information on outdoor air quality, and help their child wear a mask, respectively. About two thirds (66.3%) felt that it would be “very expensive” or “extremely expensive” to keep the air inside their house clean. Participants reported both positive and negative feelings about wearing masks, and 44.7% reported some stigma in the community about mask-wearing. Half of the participants (51.8%) reported that “some” people in their community do take action to avoid WFS exposure and 10.1% reported that “almost everyone” does.

Respondents described past impacts of WFS on themselves and their children (Table 4). Most parents (69.8%) reported their child had “a little” or “a lot” of health problems during smoke events. Common symptoms were burning eyes (66.8%), coughing (62.3%), headaches (61.8%), and difficulty breathing (48.7%). Most experienced impacts on daily life: 36.2% of child stayed home from daycare or school, and 24.1% of parents skipped work at least once. Many schools responded to smoke episodes: 57.2% modified activities during smoky days, and 29.7% disseminated WFS information to families. However, 24.1% of parents reported no action from their child’s school or daycare.

We characterized preferred sources of information and interest in future assistance with WFS exposure (Supplemental Figs. 3 and 4). There were several preferred sources of information about WFS, including local news on TV or online (49.7%), healthcare providers (45.7%), social media (42.2%), the Yakima County Health District (38.2%), and their child(ren)’s school (37.7%). Parents said they’d be “likely” or “highly likely” to utilize these resources in the future if available: WFS information social media (74.6%); free N95 masks with demonstrations (71.3%); assistance to purchase air cleaners (69.0%); written information provided by schools (67.9%); and events at a child’s school or daycare (63.7%).

**Figure 3:**
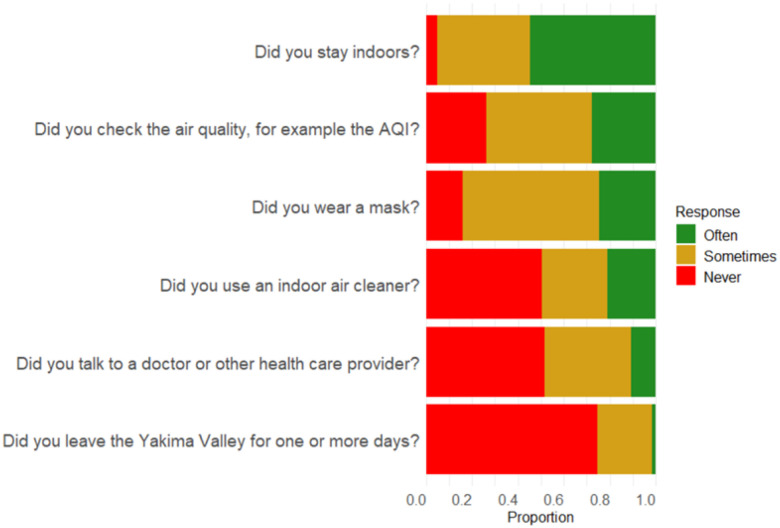
Frequency of protective actions taken in past wildfire smoke events. Respondents were asked how frequently they’ve taken each of six protective actions in past smoke events: *often*, *sometimes*, or *never*. Proportions of each response are displayed in the figure (N=199 respondents)

Participants reported protective actions taken during past WFS events (Fig. 3) and reason(s) they did not act if relevant. About half (54.3%) “often” stayed indoors during WFS events; 50% of those who did not reported that the barrier was having to go to work. Only 24.6% “often” used masks during WFS events; reported barriers to not doing so included believing they would not help (25%) and “other reason (40.6%). Few (10.7%) “often” talked with a health provider during smoke events. Of those that did not, reported barriers included feeling that it wouldn’t help (28.4%), not enough time (14.7%), no provider (10.8%), and/or it was too expensive (8.8%). About a quarter (27.8%) reported “often” checking air quality, while 46.2% reported that they did not know about the AQI. Most respondent (74.7%) never left town to avoid smoke. Barriers included having to work (25.7%) and that it was too expensive (16.6%). Half (50.3%) of parents “never” used an indoor air cleaner. Reasons for not doing so were: the expense (35.0%), uncertainty about where to purchase one (29.0%), and/or what type to get (24.0%).

### 3.3 Analysis of predictors of protective behaviors

Supplemental Table 2 displays the pairwise Pearson correlation coefficients amongst all predictors. The two types of knowledge scores (general knowledge and “how to” knowledge) were strongly correlated (rho = 0.827). Moderately strong correlations were evident for pairs of several health-related factors, including number of parent’s own existing health conditions, number of child(ren)’s existing health conditions, number of symptoms experienced by parent and/or child(ren) in past smoke events, and impact on child health in prior smoke events (rho = 0.447 to 0.580). Level of confidence to take protective actions in the future was moderately correlated with general knowledge score and “how to” knowledge score (rho = 0.398 and 0.423, respectively). Other pairs were weakly or very weakly correlated (rho < 0.3).

#### 3.3.1 Associations with total protective action score (primary outcome)

**Figure 4:**
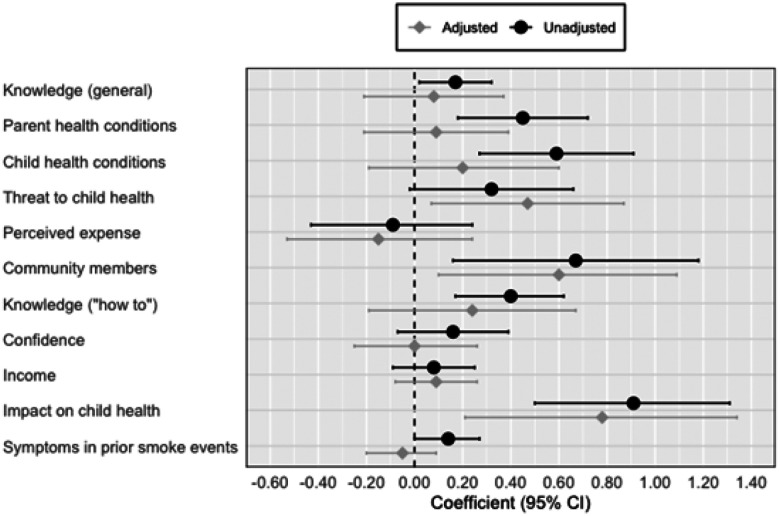
Analysis of N=11 potential predictors of protective actions. Each predictor was standardized in the study sample (N=199) as a z-score, and associations with the total protective action score were estimated in linear regression with robust standard errors. The main models were unadjusted for other factors; secondary analyses included adjustment for all other predictors. Coefficients represent the difference in protective action score associated with a 1-unit increase in the z-score of each predictor.

Total action scores ranged from 6 to 18, with a mean (SD) of 11.1 (2.2). Associations between each hypothesized predictor and total protective action score are presented in Fig. 4. In bivariate analyses, the factors which exhibited the strongest associations with total action scores were impact on child health in prior smoke events (ß=0.91; 95%CI: 0.51, 1.31); number of community members taking action against wildfire smoke exposure (ß =0.67; 95%CI: 0.16, 1.18); number of child(ren)’s existing health conditions (ß =0.59; 95%CI: 0.27, 0.91); number of parent’s own existing health conditions (ß =0.45; 95%CI: 0.18, 0.72); “how to” knowledge score (ß =0.40; 95%CI: 0.17, 0.62); general knowledge score (ß =0.17; 95%CI: 0.02, 0.32); and number of symptoms experienced by parent and/or child(ren) in past smoke events (ß =0.14; 95%CI: 0.00, 0.27). Other factors were associated with the total action score in the expected direction, including perceived threat of wildfire smoke to child health, expected expense of keeping indoor air clean, and confidence to take protective actions, but associations were small and/or imprecise. Income was not associated with the total action score.

The results of sensitivity analyses were largely consistent with unadjusted bivariate models (the primary approach). With mutual adjustment of potential predictors, most associations were attenuated, though the direction of associations were largely the same as those observed in unadjusted models. One difference is that the relationship between perceived threat of wildfire smoke to child health and total action score became stronger and more precise with adjustment for all factors (ß_adjusted_=0.47; 95%CI: 0.07, 0.87). The number of community members taking protective actions, “how to” knowledge score, and the impact on child health in prior smoke events again exhibited relatively strong associations with total action score. Significant factors identified through stepwise variable selection were consistent as well. The final model generated by stepwise selection had an R-squared of 22.7%.

#### 3.3.2 Associations with specific protective action scores (secondary outcomes)

In secondary analyses, we explored associations between predictors and frequency scores for each of the six individual protective actions in unadjusted regression models (Supplemental Table 3). The associations between possible predictors and frequency of reported actions varied by the type of protective action.

The frequency of staying indoors was associated only with count of child health conditions (ß = 0.08; 95%CI: 0.00–0.16) and perceived threat of WFS to child health (ß =0.09; 95%CI: 0.00, 0.16). Mask wearing was lower for participants reporting more negative feelings about wearing masks (ß = −0.17; 95%CI: −0.32, −0.01) and higher for those who observed other community members taking protective actions (ß = 0.15; 95%CI: 0.00, 0.30). Talking to a health care provider was linked to several factors, including the count of child health conditions (ß = 0.22; 95%CI: 0.13, 0.32), respondent health conditions (ß = 0.10; 95%CI: 0.02, 0.18), perceived threat of WFS (ß = 0.13; 95%CI: 0.01, 0.25), number of community members taking protective actions (ß = 0.22; 95%CI: 0.07, 0.38), “how to” knowledge score (ß = 0.08; 95%CI: 0.01, 0.14), WFS impact on health in WFS events (ß = 0.30; 95%CI: 0.18, 0.43), and count of symptoms in past smoke events (ß = 0.04; 95%CI: 0.00–0.08). The frequency of checking the AQI was associated with knowledge scores (both general [ß = 0.08; 95%CI: 0.02, 0.13] and “how to” knowledge [ß = 0.15; 95%CI: 0.07, 0.23]) and household income (ß = 0.07; 95%CI: 0.02, 0.12). Leaving the region was associated only with number of community members taking protective actions (ß = 0.16; 95%CI: 0.03, 0.28). Air cleaner use was associated with count of child health conditions (ß = 0.16; 95%CI: 0.04, 0.27), respondent’s health conditions (ß = 0.17; 95%CI: 0.08, 0.26), knowledge scores (both general [ß = 0.08; 95%CI: 0.03, 0.13] and “how to” [ß = 0.17; 95%CI: 0.09, 0.25]), the impact on child health in prior smoke events (ß = 0.34; 95%CI: 0.19, 0.49), number of symptoms experienced in past smoke events (ß = 0.06; 95%CI: 0.01, 0.10), and confidence in using air cleaners (ß = 0.20; 95%CI: 0.00, 0.39).

## 4. DISCUSSION

Despite the well-established impacts of wildfire smoke on child health, research on interventions for prenatal and early-life WFS exposure is lagging. We aimed to address a critical knowledge gap limiting intervention development by identifying the barriers and facilitators of WFS protective actions in communities with vulnerability to WFS events. Applying community-engaged research methods, we characterized the experiences of families in past WFS events in the Yakima Valley, an agricultural, predominantly Hispanic/Latino community, and one of the most vulnerable counties in the US Northwest, based on a community health vulnerability index for wildland fire smoke exposure.^42^ Participants reported significant impacts of past smoke events as well as high concern about the adverse effects of wildfire smoke on perinatal and child health. Many parents expressed high interests for more resources to better protect their children from WFS exposure in the future. Some protective actions, such as staying indoors, were relatively common, but frequencies varied substantially across types of action. Overall, we identified substantial opportunity for increasing exposure reduction behaviors in the future, which could ultimately mitigate harmful effects of WFS on child health. Analyses of possible predictors suggested specific barriers to action, including lack of “how to” knowledge, and also community strengths that promoted protective behaviors. These findings could be directly applied to developing feasible and acceptable intervention protocols in this and similar communities.

This study provides unique insight into community members’ experiences during acute WFS events, expanding the sparse literature on WFS perceptions, frequency of individual-level protective actions, and predictors of protective actions.^19, 43–44^ There are some similarities between our findings and others. We observed high rates of health symptoms experienced by adults and children during WFS events, qualitatively consistent with those reported in a large study of the pediatric health impacts of the 2003 Southern California wildfires, in which 29% of children reported at least one respiratory symptom, such as coughing or wheezing, and 48% reported eye irritation, itchiness, or watering.^45^ Parents in Spain in 2012 reported increased coughing, sore throat or eye problems for their children, though rates were lower than in our sample.^46^ In our study we observed strong concerns about the dangers of WFS, higher than that recorded in studies of general adult populations,^47^ though direct comparisons are difficult due to differences in the way questions were asked. One qualitative study of parents in fire-prone regions of Australia found that parents worried about dangers of the fires rather than the smoke from fires,^48^ even though the population-level public health impacts of wildfire smoke is much more harmful than that of the fires themselves.^49^

We assessed parents’ knowledge and preferred sources of WFS information because information dissemination is a core component of public health assistance to wildfire-prone communities. While parents in our study demonstrated a fair amount of knowledge about WFS health risks and methods of self-protection, many had uncertainty or misperceptions, indicating that increasing information and educational campaigns in this community may be needed. Several studies have investigated the prevalence, content, uptake and utility of WFS risk communications in various wildfire-prone settings.^19, 50–53^ A recent review of WFS communications included findings from 26 individual studies and identified several limitations of current communication efforts, including lack of content on approaches to reduce exposure in messaging and inadequate input from vulnerable populations.^20^ For example, an analysis of WA State communications about WFS showed that most messaging did not include any guidance about how to take protective actions.^52^ In our sample, participants reported that their preferred source of information include healthcare providers, local news on TV or online posts. This is consistent with other research highlighting the importance of local sources of information, such as those from local and tribal organizations, rather than information disseminated by state or federal agencies.^50^ Partnering with potential local bilingual news outlets, like Univision Yakima Tri-Cities or Radio KDNA, a local bilingual radio station, could be effective for outreach to Yakima Valley families, including farmworkers.

Importantly, we found that many families are taking at least one protective action during WFS events, but many recommended actions were only taken sometimes, if at all. Other studies similarly observed mixed rates of engagement in protective behaviors during smoke events, including mask wearing,^19, 54–55^ use of indoor air cleaners,^19,44,56^ staying indoors,^45,55,57^ and consulting local air quality data.^57^ Some of these were conducted in vulnerable communities,^55,58–59^ but almost none focused on early-life WFS exposures.^60^ We observed several significant predictors of actions, which can be compared to the small number of prior studies on these relationships. Some found that higher socioeconomic status is associated with use of indoor air cleaners,^19,61–62^ yet household income and perceptions about expensiveness did not predict frequency of air cleaner use here. Consistent with others, knowledge about WFS, especially practical “how to” knowledge, was associated with protective actions^19,60,63^

One striking and consistent finding about predictors of overall protective actions relates to social norms and community cohesiveness. Our analysis revealed that observing other community members taking preventative actions was one of the strongest predictors of adoption of protective behaviors. This social modeling effect aligns with research on social influence during disasters.^64^ Interviews and group discussions in WFS-impacted areas in WA and CA have highlighted the importance of community-centered public health action and social norms.^58–59^ Notably, the concept of *familismo*, prominent in Latino communities such as the Yakima Valley, provides a valuable framework for understanding our results. *Familismo* emphasizes strong family bonds, collective decision making, and mutual support extending beyond immediate family to broader social networks.^65^ This cultural value creates natural channels for information dissemination about protective measures against WFS. Future interventions could strategically leverage these existing social structures by identifying and recruiting community champions who will model protective behaviors, organizing demonstration events at community gatherings, and developing family-centered messaging that resonates with cultural values.

Several key strengths of our study enhanced the rigor of its findings. The application of the RANAS framework provided a strong theoretical foundation for survey development and the analytic approach, enabling a multidimensional understanding of the potential factors, including knowledge, confidence, threat appraisal, beliefs about costs, and social norms, that could motivate protective health behaviors during wildfire smoke events.^59^ This framework also provides clear guidance on behavior change techniques associated with each factor; this facilitates translation from observation to interventions aimed at motivating individual-level actions. The focus on parents and early-life exposures in a vulnerable community is unique, as the limited research on parental perceptions of WFS to date has been conducted in relatively high socioeconomic populations.^60,66^ Additionally, the community-engaged approach, including the active participation of bilingual undergraduate students who belong to the Yakima Valley community, aided in building trust among locals, increased the cultural and literacy accessibility of survey tools, and drove high community engagement and participation. Notably, many participants expressed high interest in local environmental health research, and the majority volunteered contact information for future research involvement. Finally, the study’s emphasis on solution-oriented questions rather than simple description of the harmful impacts of WFS on public health increases the translational potential.

Several limitations should be considered when interpreting these findings. First, participants were invited to our study using convenience sampling. While the sociodemographic characteristics of our study sample was similar to those of vulnerable Yakima Valley community member, individuals who elected to participate may be more interested in WFS and concerned about impacts on their children than those who did not. Therefore, the rates of protective actions we observed may be overestimates of the true frequencies, particularly for healthcare consultation and air cleaner use, which showed the strongest associations with health concerns, and associations between predictors and reported actions may not reflect relationships in the underlying population. Second, literacy posed a barrier for some participants, potentially affecting survey comprehension and response accuracy, though most participants took the survey in-person and could request assistance from the study team. Reviews of survey responses indicated a high degree of internal consistency, suggesting that comprehension was not a significant limiting factor. Third, our sample size was relatively small, and we lacked statistical power to detect associations that were small in magnitude. Despite this, we observed several significant associations between hypothesized predictors and total action scores. Fourth, we limited length and complexity of the survey out of consideration for participant burden, and we could not explore all potential predictors of protective actions. Also, our assessment of actions was limited: for example, we asked about mask usage but not type of masks used, though masks vary in effectiveness,^67^ and we assessed whether parents consulted local air quality data but not how they used the information.^60^ Finally, it is difficult to infer causality based on observed associations in a cross-sectional study, and we could not account for temporal relationships between predictors and reported protective behaviors. Despite these limitations, the consistency of observed patterns with existing literature on health behavior suggests our findings provide meaningful insights into protective behavior adoption

## 5. CONCLUSIONS

This study contributed to the growing science on WFS and child health with a solution-oriented lens on the experiences of families during smoke episodes. We present novel data on the impacts of WFS on parent and child health, perceptions towards WFS risks, interests in future resources for WFS self-protection, WFS knowledge (general and practical), and predictors of protective actions in a community highly-impacted by wildfire smoke. To our knowledge, this is the first such investigation in a vulnerable community with a focus on child health, expanding a very limited literature on these topics in any study population. Ongoing and future work will directly apply these findings to intervention development and evaluation in full-scale clinical trials and implementation research.

## Supplementary Material

This is a list of supplementary files associated with this preprint. Click to download.

- SUPPLEMENTALMATERIALScopy.docx

## Figures and Tables

**Table 1:** Description of the study population, consisting of N = 199 parents of children in the Yakima Valley.

Participant characteristics^a^	N (%) or Mean (SD)
**Age (years)**	36.2 (8.7)
Missing	3 (1.5)
**Gender**	
Female	176 (88.4)
Male	22 (11.1)
Missing	1 (0.5)
**Reported race and ethnicity (Select all)**	
American Indian or Alaskan Native	10 (5.1)
Asian	5 (2.5)
Black or African American	5 (2.5)
Hispanic or Latino	167 (84.8)
Middle Eastern or North African	1 (0.5)
Native Hawaiian or Pacific Islander	0 (0.0)
White	19 (9.6)
Missing	2 (1.0)
**Country of birth**	
US	85 (43.1)
Mexico	109 (55.3)
Other	3 (1.5)
Missing	2 (1.0)
**Length of residence in study area (years)**	
2–3	18 (9.0)
4–5	16 (8.0)
6–9	23 (11.6)
10+	142 (71.4)
**Number of children in household**	2.5 (1.3)
Missing	6 (3.02)
**Access to affordable healthcare for respondent**	
Yes	136 (68.7)
No	39 (19.7)
Not sure	13 (6.6)
Prefer not to respond	10 (5.1)
Missing	1 (0.5)
**Access to affordable healthcare for child**	
Yes	171 (86.4)
No	8 (4.0)
Not sure	9 (4.5)
Prefer not to respond	10 (5.1)
Missing	1 (0.5)
**Highest level of education completed**	
No schooling/never attended	6 (3.0)
8th grade or less	50 (25.1)
Some high school (HS) but no degree	18 (9.0)
HS or GED	60 (30.2)
Some college but no degree	21 (10.6)
Associates degree/Technical school (AA, AS)	11 (5.5)
Bachelor’s degree (BA, BS)	24 (12.1)
Master’s degree (MA, MS, MEd, MSW, MBA, MPH)	5 (2.5)
Professional or Doctorate degree (PhD, EdD, MD, JD)	0 (0.0)
Prefer not to respond	4 (2.0)
**Annual household income**	
Less than 5K	18 (9.0)
5–10K	15 (7.5)
10–20K	20 (10.1)
20–30K	25 (12.6)
30–40K	39 (19.6)
40–50K	22 (11.1)
50–75K	34 (17.1)
75–100K	9 (4.5)
100–200K	3 (1.5)
> 200K	0 (0.0)
Prefer not to respond	14 (7.0)
**Participation in programs by household members (Select all)**	
Food stamps or SNAP	82 (41.4)
WIC programs	74 (37.4)
Free or reduced price lunch	120 (60.6)
None of these	27 (13.6)
Prefer not to respond	7 (3.5)

a. If not listed, there were no instances of missing data. Abbreviations: GED = General Education Development; AS = Associate of Science; BS = Bachelor of Bcience; BA = Bachelor of Arts; MA = Master of Arts; MS = Master of Sciences; MEd = Master of Education; MSW = Master of Social Work; MBA = Master of Ausiness Administration; MPH = Master of Public Health; PhD = Doctor of Philosophy; EdD = Doctor of Education; MD = Doctor of Medicine; JD = Juris Doctor; SNAP = Supplemental Nutrition Assistance Program; WIC = Women Infants and Children

**Table 2:** Reported health conditions, health behaviors, outdoor employment, and indoor home environment

Reported health, health behavior, and other environmental factors^a^	N (%)
	Total N = 199
**Existing health conditions of parent (Select all)**	
**Asthma**	**33 (16.6)**
**COPD**	**1 (0.5)**
**Other respiratory disease**	**4 (2.0)**
**High blood pressure**	**22 (11.1)**
**Other heart disease**	**3 (1.5)**
**Type II diabetes**	**20 (10.1)**
**Anxiety or depression**	**52 (26.1)**
**Allergies related to lungs, eyes, or nose**	**45 (22.6)**
**Skin problems**	**27 (13.6)**
**None of the above**	**91 (45.7)**
**Existing health conditions of child(ren) (Select all)**	
**Asthma**	**58 (29.6)**
**Allergies**	**77 (39.3)**
**Eczema**	**42 (21.4)**
**Diabetes**	**2 (1.0)**
**Heart condition**	**4 (2.0)**
**None of these**	**94 (48.0)**
**Missing**	**3 (1.5)**
**Current smoking status of parent**	
Yes	8 (4.0)
No	190 (96.0)
Missing	1 (0.5)
**Household smokers (other than respondent)**	
Yes	21 (10.7)
No	175 (89.3)
Missing	3 (1.5)
**Indoor smoking (if any smoker in household, N = 24)**	
No smoking indoors	17 (70.8)
Yes; occurs less than once a week	3 (12.5)
Yes; occurs at least once a week	1 (4.2)
Missing	3 (12.5)
**Job requires outdoor work (respondent)**	
Yes	74 (37.4)
No	124 (62.6)
Missing	1 (0.5)
**Average number of hours working outdoors during summer and fall (if outdoor job reported, N = 74)**	
<2 h	5 (6.9)
2–4 h	9 (12.0)
4–8 h	32 (44.4)
>8 h	26 (36.1)
**Reliable air conditioning in home**	
Yes	137 (69.5)
No	41 (20.8)
Sometimes	19 (9.6)
Missing	2 (1.0)

a. If not listed, there were no instances of missing data. Abbreviations: COPD = chronic obstructive pulmonary disease

**Table 3:** Reported perceptions about wildfire smoke risks, impacts, behaviors and preparedness for future events

Survey question and response categories^a^	N (%)
	Total N = 199
**How much do you view wildfire smoke as a threat to the health of your children?**	
**Not a threat at all**	**1 (0.5)**
**A little bit of a threat**	**6 (3.1)**
**A moderate threat**	**33 (16.8)**
**Very much a threat**	**43 (21.9)**
**An extreme threat**	**113 (57.7)**
**Missing**	**3 (1.5)**
**How confident is the parent in setting up and use a portable air cleaner in the home?**	
**Not confident at all**	**19 (9.5)**
**Somewhat confident**	**127 (63.8)**
**Very confident**	**53 (26.6)**
**How confident is the parent in checking current information on outdoor air quality?**	
Not confident at all	34 (17.3)
Somewhat confident	105 (53.3)
Very confident	58 (29.4)
Missing	2 (1.0)
**How confident are you in helping your child wear a mask to effectively reduce smoke exposure?**	
Not confident at all	13 (6.6)
Somewhat confident	102 (51.5)
Very confident	83 (41.9)
Missing	1 (0.5)
**How much do you think it would cost to keep the air inside your house clean during wildfires?**	
Not expensive at all	0 (0.0)
A little expensive	9 (4.5)
Somewhat expensive	58 (29.1)
Very expensive	51 (25.6)
Extremely expensive	81 (40.7)
**How do you feel about wearing masks when the air is smoky due to wildfires? (Select all)**	
I like wearing masks	54 (27.1)
I will wear one for safety, but I don’t like wearing it	108 (54.3)
They are uncomfortable	58 (29.1)
It is not convenient to wear a mask	11 (5.5)
Masks are not helpful in reducing smoke exposure	12 (6.0)
It is hard to know what type of mask to wear	40 (20.1)
Masks are too expensive	18 (9.0)
I want to wear masks but sometimes cannot get them	26 (13.1)
None of the above	3 (1.5)
**If you wear a mask when it is smoky outdoors, would your friends, family, or co-workers disapprove?**	
No, not at all	110 (55.3)
Some might disapprove	74 (37.2)
Yes, many would disapprove	15 (7.5)
**How many people in your community take action to avoid smoke exposure?**	
Very few	75 (38.2)
Some	103 (51.8)
Almost everyone	20 (10.1)

a. If not listed, there were no instances of missing data.

**Table 4:** Reported experiences of parent and child(ren) in past wildfire smoke events

Survey question and response categories	N (%)
	Total N = 199
**Do any of your children experience health problems when it is smoky outside?**	
**None**	**61 (31.3)**
**A little**	**91 (47.7)**
**A lot**	**43 (22.1)**
**Missing**	**4 (2.0)**
**Do you or your child(ren) experience any of the following health effects during wildfire smoke days? (Select all)**	
**Difficulty breathing**	**97 (48.7)**
**Coughing**	**124 (62.3)**
**Burning eyes**	**133 (66.8)**
**Runny nose**	**87 (43.7)**
**Headache**	**123 (61.8)**
**Sore throat**	**87 (43.7)**
**Itchy skin**	**50 (25.1)**
**Poor sleep**	**52 (26.1)**
**Feeling sad or anxious**	**50 (25.1)**
**None of the above**	**18 (9.0)**
**Did wildfire smoke ever impact you or your child(ren) in any of these ways? (Select all)**	
I skipped work	48 (24.1)
A child stayed home from daycare or school	72 (36.2)
My child or I went to a clinic or hospital because of health problems	28 (14.1)
We had to leave the area for a town with cleaner air	19 (9.5)
None of the above	80 (40.2)
**Has the daycare or school ever taken actions on wildfire smoke?**	
Sent information to parents about wildfire smoke	43 (29.7)
Modified school activities on smoky days	83 (57.2)
Cancelled school on smoky days	31 (21.4)
Other	4 (2.8)
No actions	35 (24.1)

## Data Availability

Data available upon request, conditional on ethics approval and completion of required data sharing agreements.

## Abbreviations

AQI: Air Quality Index
CAB: Community Advisory Board
CEnR: Community Engaged Research
HIS: Hispanic Serving Institution
IRBs: Institutional Review Boards
Tribal Institution–NASNTI: Native American Serving Non
PM_2.5_: Particulate Matter 2.5
REDCap: Research Electronic Data Capture
regulation–RANAS: Risks, Attitudes, Norms, Abilities and Self
SNAP: Supplemental Nutritional Assistance Program
UW: University of Washington
WFS: Wildfire Smoke
WIC: Women, Infants and Children
